# Reversion of Hormone Treatment Resistance with the Addition of an mTOR Inhibitor in Endometrial Stromal Sarcoma

**DOI:** 10.1155/2014/612496

**Published:** 2014-07-08

**Authors:** J. Martin-Liberal, C. Benson, C. Messiou, C. Fisher, I. Judson

**Affiliations:** The Royal Marsden Hospital, Fulham Road, London SW3 6JJ, UK

## Abstract

*Background*. Endometrial stromal sarcomas (ESS) are a subtype of gynaecological sarcomas characterized by the overexpression of hormone receptors. Hormone treatment is widely used in ESS but primary or acquired resistance is common. The mammalian target of rapamycin (mTOR) pathway has been suggested to play a key role in the mechanisms of hormone resistance. Recent studies in breast and prostate cancer demonstrate that this resistance can be reversed with the addition of an mTOR inhibitor. This phenomenon has never been reported in ESS. *Methods*. We report the outcome of one patient with pretreated, progressing low grade metastatic ESS treated with medroxyprogesterone acetate in combination with the mTOR inhibitor sirolimus. *Results*. Partial response was achieved following the addition of sirolimus to the hormone treatment. Response has been maintained for more than 2 years with minimal toxicity and treatment is ongoing. *Conclusion*. This case suggests that the resistance to the hormone manipulation in ESS can be reversed by the addition of an mTOR pathway inhibitor. This observation is highly encouraging and deserves further investigation.

## 1. Introduction

Sarcomas are a heterogeneous group of more than 50 different malignancies characterized by their poor prognosis and the lack of effective treatments. They can arise anywhere in the body, and the uterus is one of the most common sites [1]. However, uterine sarcomas are rare and they constitute only 1% of female genital cancer and approximately 3–5% of all uterine malignancies [2]. Endometrial stromal sarcomas (ESS) account for approximately 10% of all uterine sarcomas [3] and they characteristically express hormone receptors (HR), that is, oestrogen (ER) and progesterone (PgR) receptors [4]. The expression of ER in ESS ranges between 40 and 80% and PgR is expressed in around 60–100% of cases [5–7]. In addition, ER and PgR expression have been positively correlated with survival in many studies [4, 8, 9]. Furthermore, it is known that uterine cell proliferation and differentiation are regulated in part by hormones. Therefore, the use of oestrogen modulation as an anticancer treatment is a rational therapeutic approach. Although there are no prospective randomised controlled trials of hormonal therapy in uterine sarcomas, a large number of studies have demonstrated its efficacy in ESS [5, 7, 10–13]. Indeed, this therapeutic strategy is widely used given that response rates to chemotherapy are low [14, 15].

ESS are not the only hormone-driven malignancies [16]. Hormones also play a key role in a number of other tumours, especially prostate [17] and breast cancer [18].

The inhibition of the aromatase enzyme in breast cancer has significantly improved the outcome of the patients with HR positive tumours [19–22]. Unfortunately, primary or acquired resistance to hormone treatment is not infrequent. Some studies suggest that this resistance might be mediated through the mammalian target of rapamycin (mTOR) pathway [23–25]. Thus, a study by Baselga et al. demonstrated that everolimus, an mTOR inhibitor, combined with an aromatase inhibitor (AI) significantly improved progression-free survival (PFS) in patients with HR positive advanced breast cancer previously treated with AI [26]. Preclinical studies showed similar results also in prostate cancer [27].

This paradigm of hormone-resistance reversibility observed in breast cancer might be valid in other hormone-driven malignancies as well. We present here the first report ever in which a patient affected by an advanced ESS with a good initial response to hormone treatment benefited from control of her disease following addition of an mTOR inhibitor upon disease progression as defined by RECIST v1.1 [28].

## 2. Case Presentation

Our patient first presented at the age of 58 years with abdominal pain. A CT scan revealed a 12 cm cystic ovarian lesion. The mass was excised and histopathological analysis showed features consistent with low grade ESS with strong ER and PgR expression. The patient had undergone a total abdominal hysterectomy (TAH) and single oophorectomy 15 years earlier due to a supposed benign condition. Subsequently to the diagnosis of ESS, pathology review of the first operation confirmed low grade ESS. Adjuvant treatment was not prescribed.

**Figure 1:**
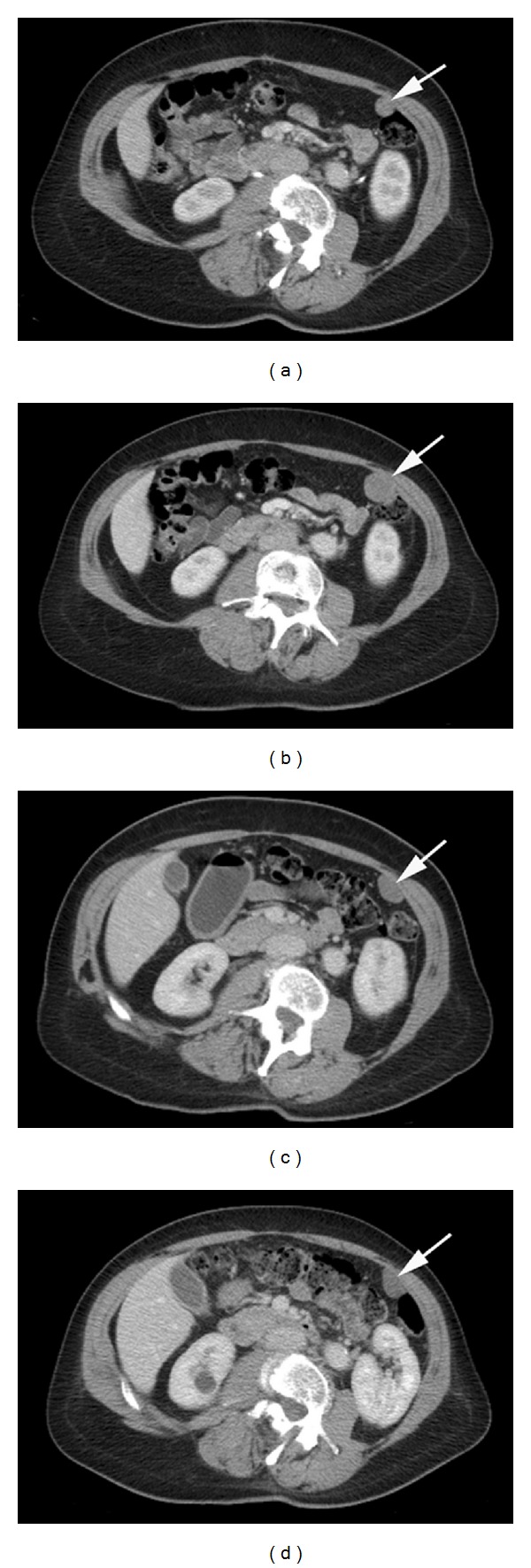
Axial contrast enhanced CT images show a peritoneal deposit within the left side of the abdomen (arrows). Prior to commencing sirolimus, the deposit progressed by RECIST 1.1 over a period of 6 months ((a) and (b)). CT staging at 4 months (c) and 13 months (d) on treatment with sirolimus showed that the deposit had reduced in size but was within the limits of stable disease by RECIST v1.1. A further pelvic deposit (not shown) also reduced in size but overall disease remained stable by RECIST v1.1. However, assessment by Choi criteria which incorporates attenuation changes classified disease status as partial response at 4 months and further partial response at 13 months.

Two years following ovarian surgery, the patient presented with right-sided abdominal pain. A new CT scan showed a 5 × 3 cm mass in the inferior pelvis and another mass of similar characteristics in the right iliac fossa. A second operation was performed and the 2 lesions were resected and the pathological analysis demonstrated relapse of her previous ESS with strong HR expression. Postoperative close surveillance and leuprorelin injections, a gonadotropin-releasing hormone (GnRH) analog, were advised. Almost 1 year later, a further relapse in the form of several peritoneal deposits and recurrence of the pelvic mass was diagnosed on a CT scan. The disease was considered unresectable so the patient started treatment with an AI, letrozole 2.5 mg once daily (od). Her disease remained stable for 4 months and the patient did not experience any significant side effects. However, a new CT scan demonstrated progression of her pelvic disease. In addition, the patient reported new abdominal discomfort. A different hormonal manoeuvre was considered and medroxyprogesterone acetate 400 mg od was started. The abdominal symptoms completely disappeared soon after starting treatment in spite of not finding significant tumour changes in regular CT scans, being classified as stable disease (SD) by RECIST v1.1. Moreover, the patient tolerated the treatment well. Nevertheless, progression by RECIST v1.1 in the dominant peritoneal nodule located anteromedial to the splenic flexure was noted after 1 year of treatment: 2.8 cm in maximum diameter compared to 1.4 cm in previous CT scan (Figure 1). The pelvic mass showed no significant changes.

In order to maximize the benefit of the hormone treatment, the mTOR inhibitor sirolimus was added to continuing medroxyprogesterone acetate in an attempt to reverse the hormone resistance. The patient started the new treatment at 3 mg od with a plan to escalate the dose depending on tolerance. However, she developed grade 3 mucositis so had to reduce the dose to 2 mg od after a short drug holiday. Unfortunately, the mucositis was still intermittently severe so she was recommended to titrate the dose of sirolimus from 1 mg od to 2 mg od depending on toxicity. With this strategy, the patient has been able to tolerate the treatment without symptoms that significantly impair her quality of life. In addition, the imaging assessments have shown renewed control of her disease. Interestingly, the first assessment CT scan performed 4 months after the addition of sirolimus demonstrated a slight reduction in size of the dominant peritoneal nodule from 2.8 cm in maximum diameter to 2.5 cm, being stable by RECIST v1.1. Furthermore, assessment by Choi criteria [29], which incorporates attenuation changes, classified disease status as partial response at 4 months and further partial response at 13 months (Figure 1). In total, the patient has been on sirolimus and medroxyprogesterone acetate for more than 2 years with acceptable tolerance and control of her disease. Her treatment is still ongoing.

## 3. Discussion

This report suggests for the first time that the resistance to the hormone treatment can be reversed by the addition of an mTOR inhibitor in other tumours apart from breast cancer. This is especially relevant in malignancies like ESS where treatment alternatives are very scarce.

The lack of effective therapeutic options makes ESS a challenging disease [30]. Although ESS tend to have an indolent course, with 5-year disease specific survival of around 90% for stages I-II and 50% for stages III-IV [31], their treatment is generally hindered by their poor responsiveness to chemotherapy [32]. Hormone treatment is considered as a valid approach depending on the hormonal status, setting (adjuvant or recurrent/metastatic), volume, and pace of the disease [2]. The maximization of the hormone treatment is crucial since these patients may benefit longer without the side effects of the chemotherapy if the hormone resistance is reversed. In breast cancer, it has been demonstrated that oestrogen-independent phosphorylation of ER-*α*, specifically on Ser167, is one of the contributing causes to development of hormone resistance, as well as a prognostic marker [33]. This phosphorylation is mediated by a substrate of mTOR complex 1 (mTORC1) called S6 kinase 1 (S6K1) [34]. Therefore, inhibition of mTOR activity is a rational therapeutic approach in order to reverse the resistance to the hormone treatment.

Our patient may be a good example of this. However, since this is a report of a single patient, the results must be taken cautiously. The very nature of low grade ESS, with indolent courses in the majority of cases, may be responsible for the long period of stabilisation of disease in our patient. However, her tumour had progressed several times before the introduction of the mTOR inhibitor suggesting that this was not the case. Moreover, hormone treatment achieved periods of disease stabilisation, indicating sensitivity to this strategy. The new long-lasting control of the disease after the addition of sirolimus to the medroxyprogesterone acetate, with sustained response by Choi criteria [29], might be a sign of the reversion of the hormone resistance.

Another point worthy of debate is the value of mTOR inhibition alone in ESS. A positive phase III trial with the mTOR inhibitor ridaforolimus as maintenance treatment in sarcomas has recently been reported [35]. The overall benefit of the treatment was an improvement of only 3 weeks in PFS. These results are clearly insufficient but indicate that mTOR inhibitors have activity in sarcomas. However, neither in that trial nor in the preceding phase II study [36] patients affected by low grade ESS were enrolled. The term “sarcomas” encompasses more than 50 different malignancies with differing molecular biology, clinical behaviour, responsiveness to treatment, and prognosis, and there is no current evidence that inhibition of the mTOR pathway is an active therapeutic strategy in low grade ESS. Therefore, the sustained response experienced by our patient may be due to the reversion of hormone resistance by sirolimus, rather than to the activity of the mTOR inhibitor alone, in parallel to breast cancer [26].

In conclusion, our case suggests that the resistance to the hormone treatment in ESS can be reversed by the addition of an mTOR pathway inhibitor. In addition, it illustrates what is increasingly being supported in non-GIST soft-tissue sarcomas, which is the use of personalized tumour response criteria involving changes in density with or without changes in size (such as Choi criteria), rather than just assess differences in tumour measurements, like RECIST [37]. These observations, although not definitive, are highly encouraging and deserve further investigation.
